# More Women Must Lead in Global Health: A Focus on Strategies to Empower Women Leaders and Advance Gender Equality

**DOI:** 10.5334/aogh.3213

**Published:** 2021-07-12

**Authors:** Amie Batson, Geeta Rao Gupta, Michele Barry

**Affiliations:** 1 WomenLift Health, Stanford Global Health, Stanford University, Palo Alto, USA; 2 3D Program for Girls and Women, United Nations Foundation, Washington, D.C., USA; 3 Center for Innovation in Global Health, Stanford University, Palo Alto, USA

## Abstract

Despite comprising 70% of the health workforce, women fill only 25% of senior and 5% of top health organization positions. Greater diversity in global health leadership, particularly greater representation of women, is essential to ensure diverse perspectives and ideas inform policies and priorities. Interviews and literature reviews surfaced many of the key challenges that women in global health face at individual, organizational and societal levels. Initiatives working to advance women’s leadership are encouraged to consider 5 key priorities that address these challenges.

While women comprise 70% of the health workforce, they remain the minority in global health leadership, filling only 25% of senior and 5% of top health organization positions [1]. In comparison to men, women in leadership positions are more likely to directly respond to the concerns of the community, to allocate funds toward education, health, and nutrition, to prioritize the needs of women, children, and marginalized groups, and to increase research on women’s health issues [2,3,4]. Over the past several months, the COVID-19 pandemic has laid bare the tremendous barriers to achieving equitable health policy decision making. While women comprise the bulk of frontline health workers, care deciders, and caregivers, they are too often missing from the rooms where health policy, funding, and research decisions are made. As a result, invaluable insights from their leadership talents and lived experiences are also excluded.

What’s holding women back? One reason is that women are the primary health care providers for families (80% in the United States). More importantly, there are implicit biases in the health industry. Research describes women in medicine and science being held to a higher standard at work than men, perceived as aggressive while their male colleagues are perceived as assertive, and channeled towards less-recognized support tasks [5]. These include well-documented gender pay gaps, with male doctors earning an average of 28% more than female doctors in the US [6].

The Center for Creative Leadership, a global leadership development organization, and WomenLift Health, a Bill & Melinda Gates Foundation-funded program promoting women’s leadership in global health, conducted 27 in-depth interviews with global health experts, senior women leaders, and mid-level female global health professionals in Rwanda, India, and the US, supplemented with literature reviews on women’s leadership development and existing leadership programs and networks. The results highlighted the continuing imbalance of representation of women compared to men in the highest health leadership positions in academia, government, and healthcare, with the number of women leaders in global health increasing by an average of just over 2% over the past decade. At work, women experienced challenges at the individual, organizational, and societal levels (described in more detail below). While these issues are not unique to the global health field, the disparities are particularly glaring given the large pool of women engaged in health. While the barriers that stand in the way of women manifest differently depending on the cultural context, there is significant overlap in the challenges faced by women leaders in health in the US, India, and East Africa. They include:

1. Individual: Tensions between home and work responsibilities, especially as career growth opportunities often coincide with family growth and travel requirements; a lack of self-belief, self-silencing, fear of risk taking, and fear of losing authenticity while exerting power;
2. Organizational: The lack of networks, mentors, and other professional development resources; harassment in the workplace; male-oriented metrics for success that skew promotions; and biased recruitment and hiring policies; and
3. Societal: Systemic gender bias in paternalistic societies in which men and women consciously and unconsciously respond to women professionals based on their ideal concept of a wife’s, mother’s, and/or daughter’s behavior, which respondents in Rwanda and India described as being polite and submissive.

Addressing the barriers constraining women leaders in global health requires not only training and equipping the individual, but also influencing the environment in which she lives and works. Initiatives working to advance women’s leadership should consider the following key priorities:

1. **Identifying training resources that target the individual-level challenges women face:** Women, in particular, struggle with self-silencing, a lack of self-confidence, and a fear of risk taking, which can contribute to the “Imposter Syndrome,” a belief that one is not qualified or deserving of their authority, power, and credibility. To support comprehensive leadership development, organizations must deliver more than technical skills; they must also cultivate leadership skills. Coaching, mentoring, creating peer networks, and enabling membership in professional associations are all critical elements to support women leaders in confidently expanding their voice, influence, and impact.
2. **Creating more inclusive and supportive cultures, processes, and systems at the organizational level:** Organizations must examine their internal systems and processes to ensure they are creating enabling environments for women. This includes policies for pay equity, paid leave, and flexible work arrangements for both men and women, as well as fostering environments where sexual harassment, discrimination, and bias are not tolerated.
3. **Promoting gender transformative policies and guidance at the societal level:** At the societal level, broader gender norms and those that govern the global health field must be changed so that women’s leadership and gender equality are valued and recognized as a requisite to addressing the world’s health challenges. High-level influencers and policymakers must be engaged to elevate the value of women’s leadership and the challenges women face in society, and to realize that overcoming existing barriers will require policies that actively sponsor women’s advancement and programs to support women’s leadership training.
4. **Working collaboratively with partners to scale impact:** Leadership training programs in global health will be more impactful if they are part of the larger movement to advance gender equality. There is a diverse ecosystem of organizations working to advance women’s leadership in global health, including WomenLift Health, Global Health 50/50, CORO India, Co-Impact, London School of Hygiene and Tropical Medicine, and Women in Global Health, along with many national organizations, universities, and initiatives. To drive meaningful change at scale, individual, organizational, and societal change agents must work together. This involves knowledge-sharing of best practices and resources, utilizing institutional networks to broaden collective networks globally, and identifying leadership opportunities across institutions.
5. **Contextualizing solutions for local environments:** There is no one-size-fits-all approach to advancing women’s leadership in global health. According to the WHO Global Health Workforce Network’s Gender Equity Hub’s published report, *Delivered by Women, Led By Men*, “there can be no universal blueprint for addressing gender equality in the health workforce” because “countries have different starting points in terms of health systems, resource levels, health worker supply, gender equality and socioeconomic context [7].” To be most effective at the national and sub-national levels, policies and solutions across all sectors, including health, must be locally led and implemented, while considering local cultural and social norms and barriers [8].

COVID-19 has underscored the positive outcomes for society when women lead. The 7% of heads of state who are women have had an outsized impact on the coronavirus pandemic. For example, both Jacinda Ardern, Prime Minister of New Zealand, and Mette Frederiksen, Prime Minister of Denmark, who as a result of combining early, decisive action informed by science with effective, empathetic engagement of their constituents, have successfully slowed the spread of the virus [9,10]. Systematic research has similarly demonstrated that countries and states with women as leaders have had fewer COVID-19 cases and deaths [11,12].

Women’s leadership in health is more than an issue of equity—it is the missing link that will help us more effectively address crises such as COVID-19 and reach our best health outcomes. The concentration of global health leadership among males has created enormous blind spots and perpetuated homogeneous thinking [13]. The complexity of the global health landscape demands that diverse ideas and perspectives are fully harnessed.

Moving forward, the world must both increase and enhance training and initiatives that expand the power and influence of women leaders. Traditional approaches are simply not enough to drive the change needed today. New approaches must include the voices, perspectives, and lived experiences of diverse women across countries, cultures, disciplines, and sectors (e.g., public, private, academia, non-profit). Only by changing current norms and demanding the inclusion of a wide range of women’s voices in leadership will we succeed in tackling the complex global health challenges the world faces.
